# ASPASIA: A toolkit for evaluating the effects of biological interventions on SBML model behaviour

**DOI:** 10.1371/journal.pcbi.1005351

**Published:** 2017-02-03

**Authors:** Stephanie Evans, Kieran Alden, Lourdes Cucurull-Sanchez, Christopher Larminie, Mark C. Coles, Marika C. Kullberg, Jon Timmis

**Affiliations:** 1 York Computational Immunology Lab, University of York, York, United Kingdom; 2 Centre for Immunology and Infection, Department of Biology and Hull York Medical School, University of York, York, United Kingdom; 3 Department of Electronics, University of York, York, United Kingdom; 4 GSK Medicines Research Centre, Stevenage, United Kingdom; Hebrew University of Jerusalem, ISRAEL

## Abstract

A calibrated computational model reflects behaviours that are expected or observed in a complex system, providing a baseline upon which sensitivity analysis techniques can be used to analyse pathways that may impact model responses. However, calibration of a model where a behaviour depends on an intervention introduced after a defined time point is difficult, as model responses may be dependent on the conditions at the time the intervention is applied. We present ASPASIA (Automated Simulation Parameter Alteration and SensItivity Analysis), a cross-platform, open-source Java toolkit that addresses a key deficiency in software tools for understanding the impact an intervention has on system behaviour for models specified in Systems Biology Markup Language (SBML). ASPASIA can generate and modify models using SBML solver output as an initial parameter set, allowing interventions to be applied once a steady state has been reached. Additionally, multiple SBML models can be generated where a subset of parameter values are perturbed using local and global sensitivity analysis techniques, revealing the model’s sensitivity to the intervention. To illustrate the capabilities of ASPASIA, we demonstrate how this tool has generated novel hypotheses regarding the mechanisms by which Th17-cell plasticity may be controlled *in vivo*. By using ASPASIA in conjunction with an SBML model of Th17-cell polarisation, we predict that promotion of the Th1-associated transcription factor T-bet, rather than inhibition of the Th17-associated transcription factor ROR*γ*t, is sufficient to drive switching of Th17 cells towards an IFN-*γ*-producing phenotype. Our approach can be applied to all SBML-encoded models to predict the effect that intervention strategies have on system behaviour. ASPASIA, released under the Artistic License (2.0), can be downloaded from http://www.york.ac.uk/ycil/software.

This is a *PLOS Computational Biology* Software paper.

## Introduction

To ensure that computational and mathematical models exhibit expected or observed biological behaviours, a process of calibration is employed to assign values to model parameters for which values are unknown. Subsequently parameter perturbation and sensitivity analysis techniques can be used to explore behaviours under different parameter value sets and draw conclusions as to which parameters have the greatest influence on model behaviour [1–3].

Computational models of biological processes, including cell signalling and metabolic pathways, biochemical reactions, and gene regulation, are increasingly being described via Systems Biology Markup Language (SBML) [4]. However, understanding the impact of the introduction of a biological intervention, such as the administration of a therapeutic drug, within a model after a specified time has elapsed is not trivial, and available SBML software tools do not currently provide sufficient capability in this area. Prior to the introduction of an intervention, a model must be calibrated such that baseline biological behaviours are established. This baseline must be achieved before parameter sweeps can be used to gain insight into the impact an intervention has on simulated biological responses. If the perturbation is applied before a baseline is reached, it remains uncertain whether behaviours are emerging as a result of the intervention, or as an artefact of model implementation.

To address the challenge of capturing and analysing biological interventions in SBML, we have developed ASPASIA (**A**utomated **S**imulation **P**arameter **A**lteration and **S**ens**I**tivity **A**nalysis), a freely available software package that is compatible with all versions and levels of SBML and that complements currently available SBML solver software and statistical packages. ASPASIA fulfils the key requirements for assessing the impact an intervention has on modelled biological behaviour, specifically: (i) establishing initial parameter values by perturbing parameters or species concentration fields using global parameter sampling techniques; (ii) the automatic production of SBML model files that sets model state to that chosen in parameter sampling; (iii) the production of a new SBML model file that summarises the characteristics of the original SBML model at a specific time-point from solver output, with the inclusion of an intervention; and (iv) sensitivity analysis of the new model files post intervention. Although we note that a variety of global sensitivity analysis techniques are available, ASPASIA includes the sampling-based latin-hypercube [5] and variance-based extended fourier amplitude sampling (eFAST) [6] sensitivity analysis techniques for providing an assessment of the impact an intervention has on model behaviour. Yet, as ASPASIA produces SBML model files from both parameter sampling and post the introduction of an intervention, these model files can be executed on any available SBML solver, removing any restriction on solver choice and permitting ASPASIA to complement other SBML solvers and statistical tools that provide additional sensitivity analysis techniques.

The SBML website (http://www.sbml.org) currently lists 263 SBML-related software packages [7], although we note that the existence of additional tools outside of this resource. An assessment of the specified tools and a SBML-supporting software led us to conclude that no package currently meets all of the requirements specified above. The approach to the introduction of interventions in ASPASIA differs from the application of the SBML ‘Event’ construct: interventions are added to new SBML model files generated from solver results obtained by executing the original model file, not added to the original model file. This assists in understanding the initial model state prior to the intervention, meaning hypotheses concerning the impact of that intervention are not skewed by differences in initial state. In addition, this approach ensures that ASPASIA-generated models are compatible with and complement all SBML solvers. Of the SBML-supporting packages that possess capacity to perform some sensitivity analysis of model behaviour (including SimBiology [8], SBML-SAT [9], AMIGO [10], SensSB [11], SloppyCell [12], and SoSlib [13], we found little capacity to perform an analysis of models that include the addition of and focus on understanding the contribution of an intervention. BioPARKIN [14] can examine events, but only those not impacting the species used in sensitivity analysis. Whereas a number of these packages introduce operating system and software dependencies, such as a requirement for commercially available MATLAB toolkits (SimBiology [8], SBML-SAT [9], AMIGO [10], and SensSB [11]) or operating system restrictions (SensSB), ASPASIA uses freely available packages and is operating system independent (we note that BioPARKIN [14] (C++/Python), SloppyCell [15] (Python), and SoSlib [16] (C) also meet this requirement, although omitting intervention-focused capability). In contrast to SensSB, SBML-SAT, and AMIGO, which provide capacity to perform global sensitivity analyses, ASPASIA generates new model files that can be run on any SBML solver, ensuring no restriction in tool choice.

We believe that ASPASIA can complement available SBML software packages to address a key deficiency in the availability of techniques for solving intervention-dependent SBML models, and we exemplify its application with an SBML model of competing transcription factors in a CD4^+^ T cell undergoing polarisation to become either a Th1 or a Th17 cell. Moreover, we show how hypotheses concerning the control of Th17-cell plasticity can be generated by using ASPASIA to identify features that must be exhibited by a hypothesised receptor to drive switching of an IL-17-secreting CD4^+^ T cell towards an IFN-*γ*-producing phenotype.

## Design and implementation

### Implementation and dependencies

Successful application of ASPASIA is dependent on the Java Runtime (version 1.6 or higher) and R Statistical environments (version 2.13.1 or greater). ASPASIA utilises the R packages spartan, lhs, XML, and graphing packages gplots and plotrix.

### Generating the ASPASIA settings file

ASPASIA requires an XML format settings file containing the information required for the applied technique, such as the location of the SBML model to use in the analysis, the location where generated SBML files should be produced, and the information of the SBML file parameters being modified. The complete list of settings file requirements is specified in S1 Table. To ease the generation of this file, we have produced an online ASPASIA settings file generator (www.york.ac.uk/ycil/software/aspasia) and exemplar settings files that can be modified as required.

### SBML-specific simulation techniques in ASPASIA

**Fig 1:**
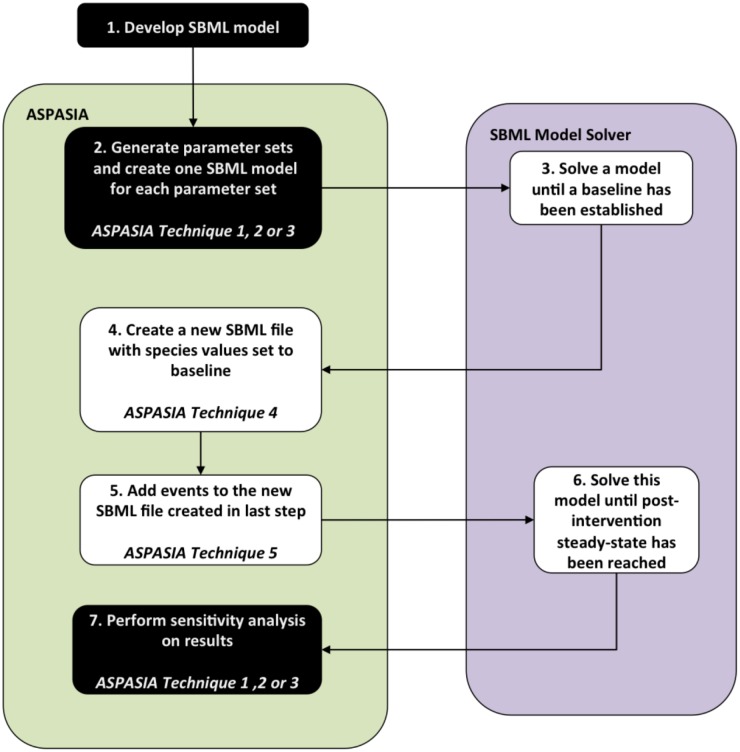
Schematic illustration of the process of applying ASPASIA to an SBML model. Using an initial SBML model as an input, ASPASIA generates a set of values as specified in a settings file, and new SBML files with parameters set to different values are created. Each model is then solved for sufficient time for a steady state to be reached and the resulting baseline values for all species supplied to ASPASIA. Then a new set of SBML files is created, and the same intervention, represented by a discrete alteration of a parameter or initial species concentration, added to all of them. The resulting files are then solved to steady state again and the effects of the intervention across the whole set of parameters and parameter values can be analysed. Black boxes represent processes that are performed only once, and white boxes represent processes that must be performed once for each model generated in step 3.

ASPASIA eases the process of calibrating and exploring SBML models of biological systems where the dynamics are dependent upon an intervention. The first stage in an intervention analysis is to run an SBML model in any solver until a determined time-point. By taking the output from the SBML solver, ASPASIA automatically creates a series of new SBML models that add an intervention to the final state that the model was in, by changing or scaling the values of specified species or parameters (Fig 1). Interventions can be added after an SBML model has been run for any specified time, or if using a solver that allows for steady state identification to be performed, when a steady state has been reached. As ASPASIA automatically detects the final time point from the output file, interventions can automatically be added to a range of models where the steady state occurs at different times. For this work presented here, models were simulated using LibSBMLSim [17] that does not have the capacity for steady state detection thus all models were run for a length of time sufficient for all populations to become stable.

#### ASPASIA techniques 1–3: SBML model files for local and global sensitivity analysis

ASPASIA produces model parameter sets where a subset of the parameters or species initial concentration levels are assigned values from a specified parameter value space. Parameter perturbation can reveal how the model behaves under different simulated conditions and identify key pathways that impact model response. As noted previously, there are a variety of sensitivity analysis techniques available (see [18] for a wider introduction to sensitivity analysis), and the choice of the algorithm should be driven by the research question being asked. By providing one local and two global sensitivity analysis techniques, with the latter two consisting of one sampling and one variance-based approach, ASPASIA provides a suitable set of tools for understanding model behaviour in a non-context dependent manner [2]. However, as ASPASIA produces SBML model files for parameter sampling and post introduction of an intervention, these model files could provide input into a number of other sensitivity analysis platforms or statistical packages if desired.

- Local Sensitivity Analysis (ASPASIA Technique 1):
  A local sensitivity analysis can reveal how robust an exhibited model behaviour is to a change in a single parameter or initial species concentration, indicating the implications that biological uncertainty or parameter estimation may have when considering the impact of model derived results [3]. ASPASIA performs a local sensitivity analysis by taking each parameter or species concentration specified in the settings file in turn as the factor of interest. This factor is assigned a value between specified minimum and maximum values, and an SBML model is file produced with the factor set at that value.
- Global Sensitivity Analysis (ASPASIA Techniques 2 and 3)
  Local analyses cannot identify compound effects where the influence of one parameter or species concentration may rely on the value of another. Such effects become evident by perturbing a number of parameters or species concentrations simultaneously, using global sensitivity analysis techniques. This approach is advantageous in revealing parameters that have a strong influence on model behaviour.
  ASPASIA Technique 2, latin-hypercube, is a sampling-based technique [5] that selects sets of parameter values where each of the parameter or species concentrations specified in the ASPASIA settings file are perturbed simultaneously, while aiming to minimise correlations between the values in each set and efficiently cover the parameter space. ASPASIA Technique 3 applies the extended Fourier Amplitude Sampling Test (eFAST) [1, 6], a variance-based technique that selects sets of parameter values from sinusoidal curves of particular frequencies through the parameter space. The sampling frequency is then used to partition variance in model response between the parameters of interest when the model results are analysed. For both techniques, the parameter space (minimum and maximum values) should be specified in the ASPASIA settings file. SBML model files are produced for each created parameter set, ensuring model responses can be generated using any SBML solver.

SBML solver responses generated for the models created in parameter sampling can be processed by ASPASIA, producing statistical and graphical output appropriate to the technique employed. The graphs in the Results section have been produced in this manner. Provided the employed SBML solver produces model output in CSV or XML format, ASPASIA provides the means to analyse the data for the included sensitivity analysis techniques described in this section.

#### ASPASIA technique 4: Creating SBML models files using SBML solver output

Technique 4 permits the creation of a new model file where parameters and species concentrations are set to values from the output of an SBMLSolver. The time at which the model files are created is defined by the user and can be the time at which steady state is reached (a time sufficient for a steady state to be reached, as in this work), or after a specific period of time, for example in the case of daily drug administration. ASPASIA creates this new model file using both the original SBML model file and the SBML solver CSV file output.

#### ASPASIA technique 5: Adding biological interventions to a steady-state SBML simulation

Utilising the SBML model set generated from an SBML model solver from Technique 4, ASPASIA provides the capacity to produce a further SBML model where one or more parameters or species concentrations are either set to a new discrete value, or to a value scaled from the final state of the SBML solver output. Perturbing values in this manner simulates how an intervention could impact the simulated biological behaviours.

## Results

### Case study

**Fig 2:**
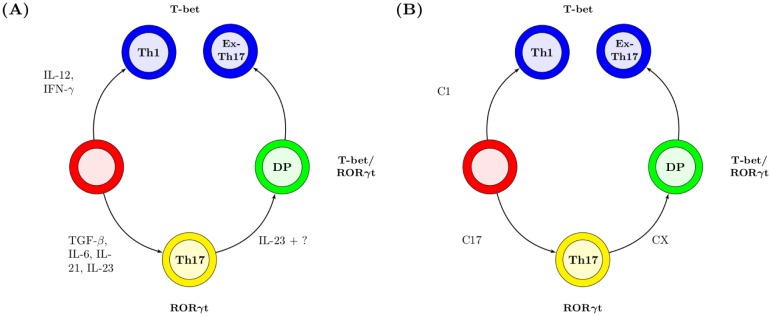
Factors controlling CD4^+^ T-cell differentiation *in vivo* and *in silico*. Upon activation, an unpolarised CD4^+^ T cell (red) can differentiate into a Th1 or a Th17 cell dependent on the cytokine milleu. Th17 cells can subsequently transition through a double-positive (DP) cell to an ex-Th17 cell. Labels on each arrow indicate cytokines involved in this process *in vivo* (A) and the model species that correspond to these cytokines *in silico* (B). In both cartoons yellow cells secrete IL-17 and IL-21, blue cells secrete IFN-*γ*, and green cells secrete a combination of IL-17, IL-21 and IFN-*γ*.

CD4^+^ T helper cells play a crucial role in the adaptive immune response following their activation through recognition of peptide/MHC complexes on antigen-presenting cells. In the presence of specific cytokines, activated CD4^+^ T cells differentiate into distinct subsets that are characterised by their cytokine-secretion profile and their expression of specific transcription factors. Thus, IL-12 drives the differentiation of Th1 cells that secrete IFN-*γ* and express T-bet, IL-4 drives the differentiation of Th2 cells that secrete IL-4 and express GATA-3, and a combination of IL-6 and TGF-*β*, along with IL-21 and IL-23 are responsible for the differentiation, maintenance and expansion of Th17 cells that secrete IL-17, IL-21, and express ROR*γ*t [19, 20]. Th17 cells have, under certain conditions, been shown to switch phenotype from an IL-17-producing Th17 cell to an IFN-*γ*-producing ex-Th17 cell that expresses T-bet and not ROR*γ*t (Fig 2A) [21, 22]. While *in vitro*-derived Th17 cells express a functional IL-12 receptor and turn on IFN-*γ* production following IL-12 stimulation [23], *ex vivo*-derived Th17 cells lack a functional IL-12 receptor [22, 24], suggesting that factors other than IL-12 are responsible for phenotype switching *in vivo*.

We have developed an SBML model to capture *in silico* the dynamics of Th17-cell plasticity *in vivo* (Fig 2B). This model can be used to infer the dynamics of a hypothetical receptor and cytokine involved in the phenotype switching of Th17 cells. The model builds on work by Yates *et al* [25] and Schulz *et al* [26] to capture the dynamics of transcription factors T-bet and ROR*γ*t in a CD4^+^ T cell undergoing polarisation and phenotype switching following exposure to exogenous cytokines. The model is freely available from the ASPASIA website, and the key equations underlying the model are listed in S2 Fig.

### Development of a computational model of Th1/Th17 polarisation

Previous models of T-cell polarisation have considered the Th1/Th2 axis, focussing on transcription factors T-bet (Th1) and GATA-3 (Th2) [25, 26]. We utilised ASPASIA to reparameterise these models to capture the dynamics of Th17 polarisation and phenotype switching, using ROR*γ*t and T-bet as markers of a particular phenotype (Fig 2B). A cell in the model is considered to be polarised if one of the transcription factors is stably expressed above baseline level. A cell that expresses both transcription factors at a higher level than at baseline is considered to be a double-positive cell.

**Fig 3:**
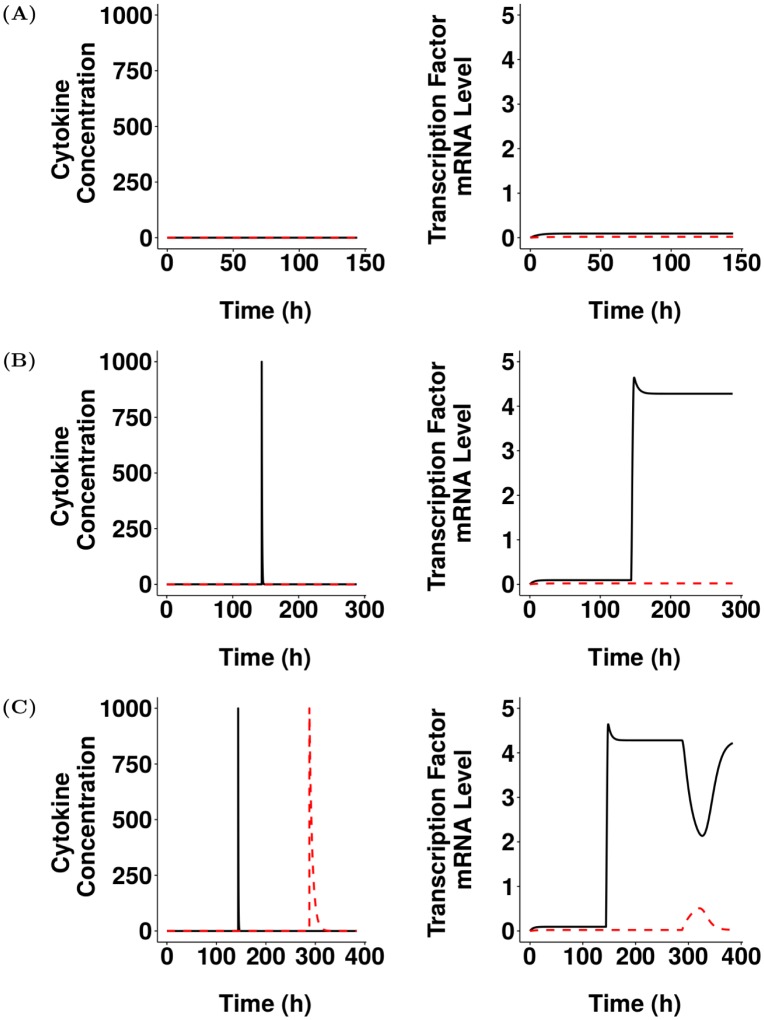
ASPASIA-generated model reflects observed biological behaviours of Th17-polarised CD4^+^ T cells. From 200 ASPASIA-generated models, a single model was selected that best captured biological behaviours. Shown are concentration of polarisating cytokines (left panels) and levels of transcription factor mRNA (right panels) in (A) the absence of type-1 polarising cytokines (C_1_) and type-17 polarising cytokines (C_17_), (B) following stimulation with C_17_, and (C) following subsequent restimulation with C_1_. Black lines represent C_17_ (left panels) and ROR*γ*t (right panels), red dashed lines represent C_1_ (left panels) and T-bet (right panels).

As many of the parameters relating to polarisation of Th17 cells were unknown, we used ASPASIA technique 2 to generate 200 unique SBML models using latin-hypercube sampling over ranges defined in S3 Fig. The models were solved using an SBML solver implementing libSBMLSim [17], with a step size of 0.12 for sufficient time until stable baseline levels of T-bet and ROR*γ*t were reached. ASPASIA techniques 4 and 5 were then used to generate an SBML model with parameters and species concentrations set to baseline values, and interventions introduced representing stimulation with either type 17 (C_17_) or type 1-polarising cytokine (C_1_) (Fig 2B). From the 200 models generated we identified the one that best captured the behaviours observed in a CD4^+^ T cell undergoing Th17 polarisation, i.e. i) no expression of either T-bet or ROR*γ*t in the absence of polarising cytokines (Fig 3A), ii) stable expression of ROR*γ*t following stimulation with type 17 polarising cytokines (Fig 3B), and iii) stability of the Th17 phenotype when introduced to type 1 polarising cytokine after initial polarisation has taken place (Fig 3C). This same model also met the criteria for a CD4^+^ T cell adopting a Th1 polarisation (S1 Table). Therefore this model was selected for further experimentation.

### Exploring the factors behind Th17-cell phenotype switching

**Fig 4:**
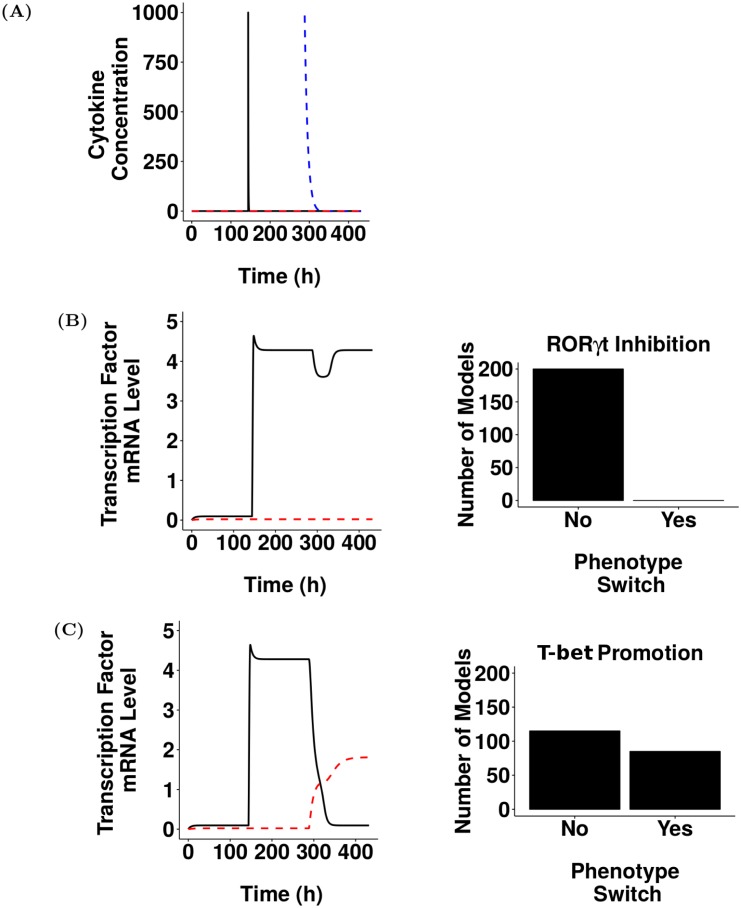
Experimentation using ASPASIA suggests that hypothetical receptor X drives phenotype switching in Th17 cells by promoting T-bet. (A) Representative profile of C_17_ (black), C_1_ (red, dashed) and C_X_ (blue, dashed) used to drive polarisation and phenotype switching. (B) Left panel: ROR*γ*t (black) and T-bet (red, dashed) expression in one representative model where C_X_ acts by inhibiting ROR*γ*t. Right panel: Number of models that have switched, or not switched under these conditions. (C) Left panel: ROR*γ*t (black) and T-bet (red, dashed) expression in a model with the same parameters as shown in left panel of B but with C_X_ acting by promoting T-bet resulting in a transition through a double-positive phase to an ex-Th17 state. Right panel: Number of models that have switched, or not switched under these conditions.

To date the biological factor(s) inducing Th17-cell phenotype switching *in vivo* is unknown. To incorporate phenotype switching of Th17 cells into the model selected in Fig 3, a hypothetical receptor that we suggest could drive phenotype switching *in vivo* was added to the model. The equations governing the dynamics of this receptor were based on a previous model of the upregulation of the IL-12 receptor during Th1 polarisation [26] and the reactions that were added to the model are shown in S4 Fig. To inform the estimation of unknown parameter values introduced by adding these reactions, we utilised values identified by Schulz *et al.* [26] and used ASPASIA technique 2 to create 200 parameter sets where the range of each parameter was specified to be 10-fold above and below the corresponding value used by Schulz *et al.* [26]. We postulated that the hypothetical receptor, namely receptor X, would drive phenotype switching of Th17 cells by either promoting T-bet or by inhibiting ROR*γ*t expression with a rate of + or − *a*_6_ respectively. Thus, two versions of the model were created from each parameter set to represent both of these possibilities S4 Fig). Each of the 400 models generated were solved with step size 0.12 for 144 hours, to allow all models to reach a steady state. ASPASIA techniques 4 and 5 were then used to add a single stimulus with C_X_, a hypothetical cytokine biding to receptor X and driving phenotype switching, and the models were solved again. Fig 4A shows a representative cytokine profile used to drive Th17 polarisation and phenotype switching by C_17_ and C_X_, respectively, in all models. In the scenario where receptor X acts to inhibit ROR*γ*t, there was an initial reduction in the mRNA level of ROR*γ*t, but once cytokine X had been removed by decay and absorption all models returned to a stable Th17 state (Fig 4B). This means that despite the dynamics of receptor signalling, and the strength of inhibition being different in every model, targeting ROR*γ*t was not sufficient to cause a phenotype switch. When C_X_ instead acted to promote T-bet, the cell switched phenotype to become T-bet expressing in 85 of the 200 models (Fig 4C). The main difference between these 85 models was the time taken for T-bet to become stable, and hence the time spent in a double positive stage (S5A Fig). In the remaining 115 models, the levels of T-bet and ROR*γ*t mRNA returned to those initially seen when the cell was in the Th17 state. These results suggest that the receptor that drives Th17 phenotype switching acts by promoting T-bet and not by inhibiting ROR*γ*t.

### Sensitivity analysis (ASPASIA technique 2)

**Fig 5:**
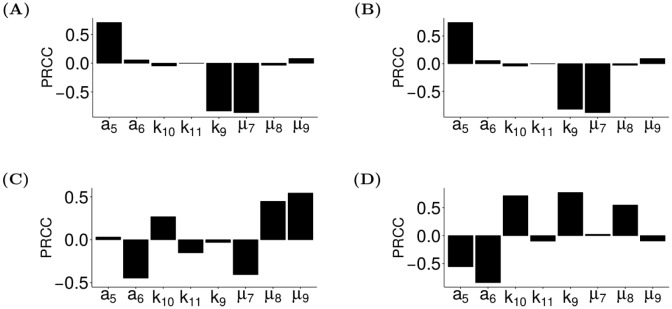
*In silico* experimentation reveals the parameters that control receptor X expression before and after polarisation with cytokines. ASPASIA was used to explore the sensitivity of the level of expression of the hypothetical receptor X following exposure to different cytokines. (A-C) Partial rank correlation coefficients (PRCC) for the correlation between receptor X expression and all parameters involved in phenotype switching were calculated for all models where receptor X acted to promote T-bet expression. PRCCs were calculated before polarisation (A), following polarisation with C_17_ (B) and after C_X_ had been introduced (C). (D) PRCC for the correlation between the time taken for the phenotype switch to occur and all parameters involved in phenotype switching for the models where a phenotype switch took place. Details and definitions of all parameters are shown in S3 Fig.

We next used ASPASIA technique 2 to perform a sensitivity analysis on the T-bet promotion model and parameter values described in S4 Fig to further examine the properties of receptor X during phenotype switching. From this analysis, we determined the parameters that were important in controlling the level of expression of receptor X at basal level, following a single round of stimulation with C_17_, and after subsequent stimulation with C_X_. Important parameters were defined as those that had the greatest correlation with the level of receptor X mRNA in each of these settings (Fig 5A–5C). Interestingly, different parameters were influential in controlling the level of receptor X before (Fig 5A and 5B) and after (Fig 5C) stimulation with C_X_. Both of these cases are potential predictors of the dynamics that a receptor driving phenotype switching must exhibit in biology. In four of the models that undergo a phenotype switch, receptor X was expressed at high level following stimulation with C_17_ alone (S5B Fig). This may suggest that a receptor that drives phenotype switching must have a high upregulation rate (*a*_5_), a low decay rate (*μ*_7_), and the level of ROR*γ*t at which formation of receptor X is at half maximum (*k*_9_) must also be low (Fig 5B). Furthermore, following stimulation with C_X_ all of the models that switched phenotype had a low level of receptor X (S5C Fig). This means that the receptor X-C_X_ complex must decay slowly (*μ*_8_) and C_X_ must also decay slowly (*μ*_9_) (Fig 5C).

For each of the models in Fig 4C where a phenotype switch occurred, we also looked at the sensitivity of how long this switch takes using ASPASIA Technique 2. The influential parameters were the upregulation rate receptor X (*a*_5_), the rate of promotion of T-bet (*a*_6_), the half-maximum level of receptor X (*k*_9_), the level of receptor X-C_X_ complex at which rate of T-bet promotion is half-maximum (*k*_10_), and the decay rate of the receptor cytokine complex (*μ*_8_) (Fig 5D). If data were available on how long a phenotype switch takes in biology, these could be used to determine the dynamics that the cytokine and receptor that drive the phenotype switch must exhibit and thus could play a role in identifying the potential receptors and cytokines driving switching. For the global sensitivity analyses shown here we note that the alternative global analysis method, ASPASIA technique 3, could also have been used.

### Sensitivity analysis to establish robustness of simulation behaviour to an intervention (ASPASIA technique 1)

**Fig 6:**
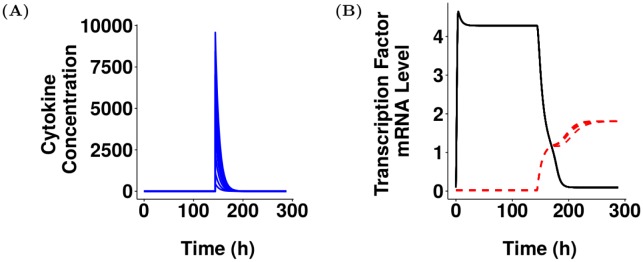
Phenotype switch is robust to changes in concentration of cytokine X. Models were polarised to a Th17 state as previously described before 10 varying concentrations of cytokine X were simulated in order to drive a phenotype switch. (A) Dynamics of cytokine X in each of the 10 models. (B) Dynamics of ROR*γ*t (black) and T-bet (red, dashed) in each of the 10 models.

We next performed an additional sensitivity analysis to determine how robust the phenotype switch was to changes in the initial concentration of C_X_. The analysis was restricted to just the 85 models in Fig 4C where a cell that was Th17 polarised switched phenotypes when stimulated with C_X_. When the intervention with C_X_ was simulated, we used ASPASIA to create 10 interventions where the initial concentration of C_X_ varied from 10 to 10000 for each model. We found that in all cases the model still switched phenotype regardless of the concentration of C_X_ applied (representative model shown in Fig 6), suggesting that the phenotype switch is extremely robust once a receptor driving switching has been introduced. This suggests that in biological systems where phenotype switching of Th17 cells does not occur, for example following infection with *Candida albicans* [21], either C_X_ and/or receptor X are not present.

### Summary: Novel mechanistic hypotheses generated using ASPASIA

We have shown how four of the techniques from the ASPASIA package have proven capable of establishing values for unknown parameters, introducing a number of biologically-inspired interventions, and conducting sensitivity analysis to understand the impact of that intervention on model response. By analysing a number of SBML models, we have been able to suggest that phenotype switching of Th17 cells is driven by the promotion of T-bet rather than the inhibition of ROR*γ*t. These conclusions have been drawn using a hypothetical model in the absence of biological data. If biological data relating to the initial and polarised levels of T-bet and ROR*γ*t in Th1 and Th17 cells had been available before model development, this would have better informed model selection prior to the addition of the hypothetical receptor to drive phenotype switching. Performing the analyses on a biologically calibrated model has the potential to assist in identifying the specific receptor driving phenotype switching of Th17 cells by highlighting the important attributes that the receptor must possess and narrowing down parameter ranges. Biological receptors that fulfil the criteria suggested via model sensitivity analysis can then be considered as potential drivers of phenotype switching, guiding further *in vivo* experimentation into receptor identification. The model ensembles and detailed instructions on how the analyses have been performed in this paper can be found on the ASPASIA website, permitting the reproduction of the approaches taken above.

## Availability and future directions

For platform independence, ASPASIA is constructed using the Java and R platforms. The ASPASIA settings file can be created using the freely available online file generator (http://www.york.ac.uk/ycil/software/aspasia). The Java executable that processes the settings file, manuals, comprehensive tutorials, and the presented case study simulation can also be downloaded from the same location. ASPASIA is open source and available under the Artistic License (2.0).

The produced SBML files are suitable for execution on a wide range of SBML-suitable platforms. ASPASIA uses solver output and the stated R packages to automatically produce statistics and plots for the included analysis techniques, easing understanding of model behaviour. We envisage adding additional sensitivity analysis techniques if appropriate. As the need for a standard to specify *in silico* experimentation becomes more critical, we intend to evaluate the support of available and upcoming experimentation specification standards (such as SED-ML [27]), potentially incorporating support of these standards in a future release. We envisage that the techniques available in ASPASIA will be very beneficial to researchers utilising other simulation methodologies outside of SBML. We hope to investigate the expansion of ASPASIA such that simulation parameter analyses can be conducted for a range of other simulation development platforms.

## Supporting information

S1 Table ASPASIA Settings file tags. Definitions of the XML tags in the ASPASIA Settings file. An example file can be downloaded from the ASPASIA website (http://www.york.ac.uk/ycil/software/ASPASIA).
(PDF)

S1 Fig ASPASIA-generated model reflects observed biological behaviors of Th1-polarised CD4^+^ T cells. The model shown in Fig 3 was examined under Th1-polarising conditions. Shown are concentration of polarisating cytokines and levels of transcription factor mRNA in the absence of type-1 polarising cytokines (C_1_) and type-17 polarising cytokines (C_17_)(A), following stimulation with C_1_ (B), and following subsequent restimulation with a C_17_ (C). Black lines represent C_17_ (left panels) and ROR*γ*t (right panels), red dashed lines represent C_1_ (left panels) and T-bet (right panels)
(EPS)

S2 Fig Equations governing processes involved in the model described in Fig 3. (PDF)

S3 Fig Ranges of all parameters used in simulation. (PDF)

S4 Fig List of additional model terms when adding a receptor to the model. (PDF)

S5 Fig Characteristics of receptor X expression in the model where T-bet promotes phenotype switching. (A) Boxplot of time taken for a phenotype switch to occur following stimulation with C_X_ (n = 84). (B) Level of receptor X prior to addition of C_X_. (C) Level of receptor _X_ following stimulation with C_X_.
(PDF)
