# Supplementary material for: ASPASIA: A toolkit for evaluating the effects of biological interventions on SBML model behaviour
Source: PLoS Comput Biol. 2017 Feb 3;13(2):e1005351. doi: 10.1371/journal.pcbi.1005351 (PMC5315406; doi:10.1371/journal.pcbi.1005351)
Supplement: S1 Table — Definitions of the XML tags in the ASPASIA Settings file. An example file can be downloaded from the ASPASIA website (http://www.york.ac.uk/ycil/software/ASPASIA). (PDF) [file pcbi.1005351.s001.pdf]

S1 Table

S1 Table: ASPASIA Settings File Tags. Definitions of the XML tags in the ASPASIA Settings file. An example file can be downloaded from the ASPASIA website (www.york.ac.uk/ycil/software/ASPASIA)

Tag	Description
sbmlFormat	Whether the model file is an SBML model. Set to true (Allows future ASPASIA extensions).
pathToSimulationParameterFile	Full path to the SBML model being processed.
parameterFileOutputFolder	Full path to folder where generated SBML models should be stored.
parameter	Information about each parameter or species concentration that is subject to an intervention or sensitivity analysis study, dependent on the technique being employed. For Robustness analysis (local), the parameter type (double or integer), minimum value, maximum value, value to use as increment in the sampling, and the parameters calibrated value, must be specified as attributes of the XML tag. The value of the tag is the parameter name:<br><parameter type=‘double’ min=‘0.01’ max=‘0.1’ inc=‘0.01’ baseline=‘0.05’>SpleenTCellArr< /parameter><br>For eFAST or Latin-Hypercube parameter sampling (global), specify the type, minimum, and maximum values:<br><parameter type=‘double’ min=‘0.01’ max=‘0.1’>SpleenTCellArr< /parameter><br>For an Intervention study, the parameter or species name is the tag value. The attributes are a double value and an intervention method, namely (in italics): (i) *replace* the value of this parameter or concentration with that in the settings file; (ii) *add* the value in the settings file to that in the SBML file; (iii) subtract the value in the settings file from that in the SBML file; (iv) *multiply* the value in the SBML file by that in the settings file; (v) *divide* the value in the model by that in the settings file:<br><parameter value=‘0.1’ method=‘multiply’>SpleenTCellArr</parameter>
Latin-Hypercube Sampling Specific Settings:	
numberparameterSamples	The number of perturbed SBML models to generate.
algorithm	Sampling algorithm to use, ’normal’ or ’optimal’. Normal selects parameter sets while attempting to minimise correlations. Optimal selects sets that entirely cover the parameter space, but the computation period is long (>48 hours for 6 parameters).
lhcPreGeneratedSampleFile	Runs the analysis with an existing parameter value set after a model file has been changed. This is the path to the parameter CSV file. No parameter information is required.
LHC Data Analysis Settings:	
pathToSolverResults	Folder where SBML solver results are located.
solverResultFileName	Name of the result file (minus csv file extension) produced by the solver for all latin-hypercube sampling experiments.
measure	Name of each simulation response measure to consider in the analysis. The name of the measure should be the tag value. The scale of that measure should be specified as an attribute. For example: <measure scale=‘microns’>displacement< /measure>
eFAST Sampling Specific Settings:	
efastCurves	Number of resample curves to employ in eFAST Sampling. See [1].
efastCurveSamples	The number of parameter values to select from each eFAST Curve.
Intervention Specific Settings:	
sbmlRunResultsFile	Path to solver output where the model has been run to steady state.
newParamFileName	Name to give the intervention containing newly generated SBML model.
