# Supplementary material for: ASPASIA: A toolkit for evaluating the effects of biological interventions on SBML model behaviour
Source: PLoS Comput Biol. 2017 Feb 3;13(2):e1005351. doi: 10.1371/journal.pcbi.1005351 (PMC5315406; doi:10.1371/journal.pcbi.1005351)
Supplement: S2 Fig — (PDF) [file pcbi.1005351.s003.pdf]

S2 Fig

List of Model Equations

C1 induced T-bet

$$\frac{s_1 \cdot C_1}{(k_1 + C_1) \cdot (1 + \frac{RORgt}{g_2})}$$

C17 induced RO-Rγt

$$\frac{s_2 \cdot C_{17}}{(k_2 + C_{17}) \cdot (1 + \frac{Tbet}{g_1})}$$

Baseline Transcription T-bet

$$b_1$$

Baseline Transcription ROR-γt

$$b_2$$

Decay T-bet

$$-\mu_1 \cdot Tbet$$

Decay ROR-γt

$$-\mu_2 \cdot RORgt$$

Decay C1

$$-\mu_3 \cdot C_1$$

Decay C17

$$-\mu_4 \cdot C_{17}$$

Auto-stimulation T-bet

$$\frac{a_1 \cdot T - bet^n}{(k_3^n + Tbet^n) \cdot (1 + \frac{RORgt}{g_2})}$$

Auto-stimulation ROR-γt

$$\frac{a_2 \cdot RORgt^n}{(k_4^n + RORgt^n) \cdot (1 + \frac{Tbet}{g_1})}$$

T-bet-induced IFN-γ

$$\frac{a_3 \cdot Tbet}{(k_5 + Tbet) \cdot (1 + \frac{RORgt}{g_3})}$$

RORgt-induced IL21

$$\frac{a_5 \cdot RORgt}{(k_6 + RORgt) \cdot (1 + \frac{Tbet}{g_4})}$$

Decay IFN-γ

$$-\mu_5 \cdot IFNg$$

Decay IL21

$$-\mu_6 \cdot IL21$$

IL21 to C17

$$k_7 \cdot IL21$$

IFNg to C1

$$k_8 \cdot IFNg$$

S2 Fig: Equations governing processes involved in the model described in Figure 3.
