# Supplementary material for: ASPASIA: A toolkit for evaluating the effects of biological interventions on SBML model behaviour
Source: PLoS Comput Biol. 2017 Feb 3;13(2):e1005351. doi: 10.1371/journal.pcbi.1005351 (PMC5315406; doi:10.1371/journal.pcbi.1005351)
Supplement: S3 Fig — (PDF) [file pcbi.1005351.s004.pdf]

S3 Fig

ID	Description	Value	Range	Data
a_1	Maximum auto-activation rate of T-bet	0.203833		[1]
a_2	Maximum auto-activation rate of RORγt	0.815603	Min: 0.00203833 Max: 2.03833	10 $\times$ either side of value for T-bet [1]
a_3	Maximum production rate of IFN-γ	0.007497877	Min: 0.00203833 Max: 2.03833	10 $\times$ either side of value for IFN-γ [2]
a_4	Maximum production rate of IL21	0.019987613	Min: 0.000203833 Max: 0.0203833	10 $\times$ either side of value for IFN-γ [2]
a_5	Maximum stimulation rate of receptor X	0.019987613	Min: 5 Max: 30	[2]
a_6	Rate of T-bet promotion or RORγt inhibition	0.304618	Min: 0.002 Max: 2	10 $\times$ either side of value for IL12 [2]
b_1	Basal transcription rate of T-bet (per min)	0.00203833		[1]
b_2	Basal transcription rate of RORγt	0.0172893	Min: 0.00203833 Max: 0.203833	10 $\times$ either side of value for T-bet [1]
g_1	Level of RORγt when transcription of T-bet is at half maximum	0.456596	Min: 0.0001 Max: 1.0	10 $\times$ either side of value for T-bet/GATA-3 [1]
g_2	Level of T-bet when transcription of RORγt is at half maximum	0.83170631	Min: 0.0001 Max: 1.0	10 $\times$ either side of value for T-bet/GATA-3 [1]
g_3	Level of RORγt when transcription of IFN-γ is at half maximum	0.625864154	Min: 0.0001 Max: 1.0	10 $\times$ either side of value for T-bet/GATA-3 [1]
g_4	Level of T-bet when transcription of IL21 is at half maximum	0.760239275	Min: 0.0001 Max: 1.0	10 $\times$ either side of value for T-bet/GATA-3 [1]
μ_1	Decay rate of T-bet mRNA	0.096016199	Min: 0.00020833 Max: 0.020833	10 $\times$ either side of value for T-bet [1]
μ_2	Decay rate of RORγt mRNA	0.18586844	Min: 0.00020833 Max: 0.020833	10 $\times$ either side of value for T-bet [1]
μ_3	Decay rate of C_1	0.1734	Decay rate of IL12	[3]

μ_4	Decay rate of C_{17}	2.038828189	Min: 0.4026<br>Max: 2.082	Range of decay rates of TGF-β, IL-6 and IL-23 [4, 5]
μ_5	Decay rate of IFN-γ mRNA	0.18586844	Min: 0.00020833<br>Max: 0.020833	10 $\times$ either side of value for T-bet [2]
μ_6	Decay rate of IL-21 mRNA	0.18586844	Min: 0.00020833<br>Max: 0.020833	10 $\times$ either side of value for T-bet [2]
μ_7	Decay rate of IL21 mRNA	0.633595	Min: 0.004026<br>Max: 2.082	10 $\times$ either side of value for IL12R [2]
μ_8	Decay rate of receptor X-C_X complex	0.527035	Min: 0.004026<br>Max: 2.082	10 $\times$ either side of value for IL12R [2]
μ_9	Decay rate of C_X	0.125304	Min: 0.004026<br>Max: 2.082	10 $\times$ either side of range for IL12, IL6 and TGF-β [2]
k_1	Level of C_1 at which T-bet transcription is at half maximum	1		[1, 2]
k_2	Level of C_{17} at which RORγt transcription is at half maximum	1		Same as T-bet [1, 2]
k_3	Level of T-bet at which T-bet transcription is at half maximum	1		Same as T-bet [1, 2]
k_4	Level of RORγt at which RORγt transcription is at half maximum	1		Same as T-bet [1, 2]
k_5	Level of T-bet at which transcription of IFN-γ mRNA is at half maximum	1		[2]
k_6	Level of ROR-γt at which transcription of IL21 mRNA is at half maximum	1		Same as T-bet [2]
k_7	Rate of conversion of IL21 mRNA into protein	0.481067361		[1, 2]
k_8	Rate of conversion of IFN-γ mRNA into protein	0.307552055		[1, 2]
k_9	Level of ROR-γt at which transcription of receptor X is at half maximum	66.7474	Min:1<br>Max: 100	$\times$ either side of value for T-bet and IL12 [2]

$\mathtt{k}_{10}$	Level of C_X-receptor X at which promotion of T-bet or inhibition of RORγt is at half maximum	2.60342	Min: 0.5<br>Max: 5	10× either side of value used by [2]
$\mathtt{k}_{11}$	Rate of formation of C_X-receptor X complex	0.545609	Min: 0.01<br>Max: 1	10 × value used by [2] for IL12R
$\mathtt{s}_1$	Rate of stimulation of T-bet by external cytokines	0.208033		[1, 2]
$\mathtt{s}_2$	Rate of stimulation of ROR-γt by external cytokines	1.35863	Min: 0.020833<br>Max: 2.08033	[1, 2]

S3 Fig: Ranges of all parameters used in the ASPASIA generated models

References

1. Yates A, Callard R, Stark J (2004) Combining cytokine signalling with T-bet and GATA-3 regulation in Th1 and Th2 differentiation: a model for cellular decision-making. Journal of Theoretical Biology 231: 181–196.

2. Schulz EG, Mariani L, Radbruch A, Höfer T (2009) Sequential polarization and imprinting of type 1 T helper lymphocytes by interferon-gamma and interleukin-12. Immunity 30: 673–683.

3. Bajetta E, Del Vecchio M, Mortarini R, Nadeau R, Rakhit A, et al. (1998) Pilot study of subcutaneous recombinant human interleukin 12 in metastatic melanoma. Clinical Cancer Research: An Official Journal of the American Association for Cancer Research 4: 75–85.

4. Kaminska B, Wesolowska A, Danilkiewicz M (2005) TGF beta signalling and its role in tumour pathogenesis. Acta Biochimica Polonica 52: 329–337.

5. Waage A, Brandtzaeg P, Halstensen A, Kierulf P, Espevik T (1989) The complex pattern of cytokines in serum from patients with meningococcal septic shock. Association between interleukin 6, interleukin 1, and fatal outcome. The Journal of Experimental Medicine 169: 333–338.
