# Supplementary material for: ASPASIA: A toolkit for evaluating the effects of biological interventions on SBML model behaviour
Source: PLoS Comput Biol. 2017 Feb 3;13(2):e1005351. doi: 10.1371/journal.pcbi.1005351 (PMC5315406; doi:10.1371/journal.pcbi.1005351)
Supplement: S4 Fig — (PDF) [file pcbi.1005351.s005.pdf]

S4 Fig

Decay $\mathbf{C_X}$

$$-\mu_9 \cdot CX$$

Decay RecX

$$-\mu_7 \cdot RecX$$

Decay $\mathbf{C_X}$:RecX Complex

$$-\mu_8 \cdot CX : RecX$$

$\mathbf{C_X}$:RecX Complex Formation

$$k_{11} \cdot CX \cdot RecX$$

RecX Formation

$$\frac{a_5 \cdot RORgt}{(k_9 + RORgt)}$$

RecX to TF

$$\frac{a_6 \cdot CX_RecX}{k_{10} + CX_RecX}$$

Where TF is either -ROR-γt or +T-bet depending on the model of plasticity under consideration.

S4 Fig: List of additional model terms when adding a receptor to the model

S4 Fig

S4 Fig
