# Supplementary material for: ASPASIA: A toolkit for evaluating the effects of biological interventions on SBML model behaviour
Source: PLoS Comput Biol. 2017 Feb 3;13(2):e1005351. doi: 10.1371/journal.pcbi.1005351 (PMC5315406; doi:10.1371/journal.pcbi.1005351)
Supplement: S5 Fig — (A) Boxplot of time taken for a phenotype switch to occur following stimulation with CX (n = 84). (B) Level of receptor X prior to addition of CX. (C) Level of receptor X following stimulation with CX. (PDF) [file pcbi.1005351.s006.pdf]

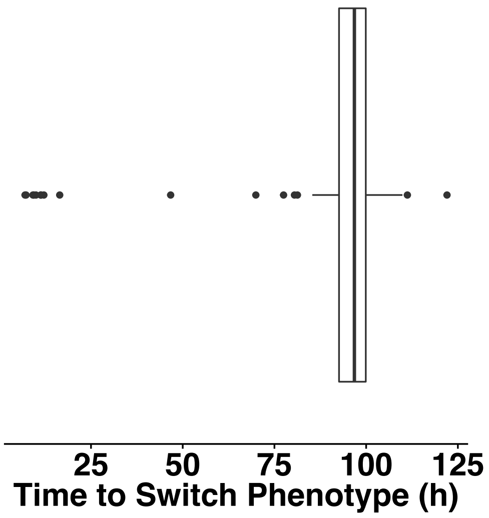

(a)

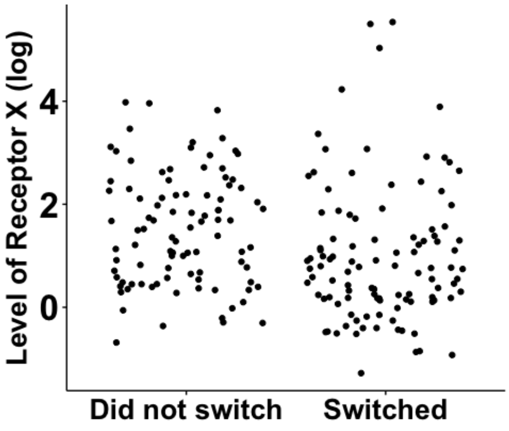

(b)

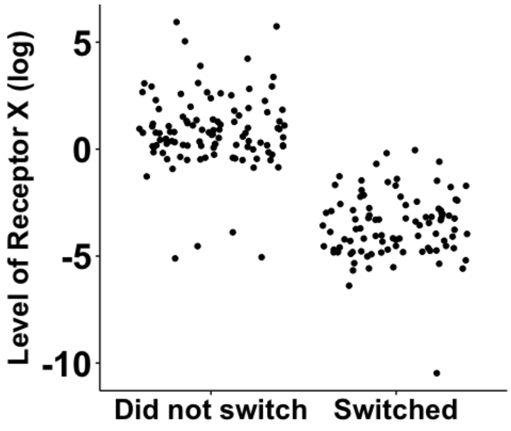

(c)

S5 Fig: Characteristics of receptor X expression in the model where T-bet promotes phenotype switching. (A) Boxplot of time taken for a phenotype switch to occur following stimulation with C_X (n=84). (B) Level of receptor X prior to addition of C_X. (C) Level of receptor $_X$ following stimulation with C_X.
